# Changing mobility patterns and road mortality among pre-license teens in a late licensing country: an epidemiological study

**DOI:** 10.1186/1471-2458-13-333

**Published:** 2013-04-11

**Authors:** Divera Twisk, Niels Bos, Jean T Shope, Gerjo Kok

**Affiliations:** 1 SWOV Institute for Road Safety Research, PO Box 1090, Leidschendam, 2260 BB, The Netherlands; 2 University of Michigan Transportation Research Institute, 2901 Baxter Road, Ann Arbor, MI, 48109-2150, USA; 3 Department of Health Behaviour & Health Education, University of Michigan School of Public Health, Ann Arbor, MI, USA; 4 Faculty of Psychology & Neuroscience, Department of Work and Social Psychology, University of Maastricht, PO Box 616, Maastricht, 6200 MD, The Netherlands

**Keywords:** Modal split, Late licensing, Early adolescence, Road risk, Moped riders, Cyclists

## Abstract

**Background:**

Whereas the safety of teens in early licensing countries has been extensively studied, little is known about the safety of pre-license teens in late licensing countries, where these teens also may be at risk. This risk exists because of the combination of a) increasing use of travel modes with a high injury risk, such as bicycles and mopeds, b) inexperience, and c) teens’ developmental stage, known to be associated with risk taking and novelty seeking, especially among males. To explore the magnitude and nature of pre-license road risk, this study analysed epidemiological data from the Netherlands, and hypothesized that in this late licensing country, ‘independent travel’ and the use of riskier modes of transport increase among pre-license teens 10 to 17 years of age, resulting in higher fatality rates, with ‘experience’ and ‘gender’ as risk modifying factors.

**Method:**

National travel and fatality data of pre-license adolescents in the Netherlands were analysed by traffic role (cyclist, pedestrian, car passenger and moped rider), and compared to a younger age group (0–9 years) and an older age group (18+ years).

**Results:**

The study of travel data showed that teens migrate from being car occupants to being users of riskier modes of transport, specifically bicycles and mopeds. This migration resulted in a strong rise in road fatalities, illustrating the importance of mobility patterns for understanding changes in road fatalities in this age group. The data further suggested a protective role of early cycle experience for young adolescent cyclists, particularly for young males. But further study into the underlying mechanism is needed to confirm this relationship. Moped risk was extremely high, especially among young males, and even higher than that of young male car drivers.

**Conclusions:**

The study confirmed the importance of changes in mobility patterns for understanding the rising road mortality when youngsters enter into their teens. The focus on fatalities has led to an underestimation of the magnitude of the problem because of the physical resilience of young adolescents that leads to high survival rates but probably also to long term disabilities. In addition, to explore the generalizability of these results, international comparisons among and between early and late licensing countries are necessary, especially in relation to moped riding as an alternative for car driving.

## Background

Worldwide, road injuries are a leading cause of death among teens, 10 to 17 years of age. The actual rates, however, differ greatly among countries [1]. One of the factors known to influence these rates is the age at which youngsters are legally allowed to drive a car. Countries that license late, that is from age 18 onwards, have generally better safety records than countries that license early, that is between ages 14 and 17 (see [2] for an overview). Whereas a wide range of studies has addressed the road risk of 14 to 17 year olds as car drivers, little is known about the road safety of *pre-license* teens between 10 to 17 of age –who, in late licensing countries, are still too young to legally hold a driving license. Although this group is not yet exposed to the high risk of car driving, the characteristic psychological and social development associated with the onset of adolescence may have a considerable influence on mobility patterns. Among the many factors that affect road safety levels, changes in mobility patterns are known to be one of the most influential [3-5]. Yet to date, studies on 10 to 17 year olds tend to focus on general themes such as deliberate risk taking and peer group influences (e.g., [6,7]), but seldom the development of mobility patterns by age and subsequent influences on road safety (e.g., [8]). To study these relationships and assess the implications for prevention strategies, the present study analyses the development of mobility patterns and road mortality by age among pre-license teens −10 to 17 year of age - in the Netherlands, where car drivers are licenced at age 18, and riders of mopeds and light-mopeds at 16. Mopeds and light-mopeds are powered two wheelers, with a maximum displacement of 50 cc for internal combustion engines and 4Kw for electric engines. Mopeds and light-mopeds differ in terms of their legal maximum speeds, which is 45 km/h for mopeds and 25 km/h for light-mopeds. Helmet wearing and holding a license are compulsory requirements only for mopeds, not light-mopeds.

Besides formal regulations on access to travel modes, the developmental stage of teens also plays a role. In developmental psychology, the age period between 10 and 17 is known as early adolescence and youngsters in this age period are known as ‘young adolescents’. Early adolescence covers roughly the period of puberty, when the bodies of children are transformed into those of sexually and physically mature adults. In addition to these physiological changes, this period is also characterized by social, emotional and cognitive changes [9,10]. Among the many changes in behaviour that have been observed for young adolescents, the two that are most prominent across cultures and that are most likely to affect mobility patterns are an increase in novelty seeking, and a shift in social attachments from the family unit toward peers [11]. Therefore, it was expected that youngsters will travel more frequently independently from caretakers compared to when they were children. Besides these psychological developments, their role in society also changes when they leave primary school and start attending secondary school. In the Netherlands, this transition will affect mobility, as the network of secondary schools is less finely-meshed than that of primary schools, resulting in a longer travel distance between home and school. Therefore, it was expected that car passenger travel would drop, independent travel would increase, and travel distances would rise in early adolescence (H1). Because in late licensing countries, young teens are not allowed to drive cars, the greater need for independent travel can thus only be met by the use of bicycles, walking, or – from age 16 onwards – the use of mopeds or light-mopeds. It is therefore expected that, compared to childhood, in early adolescence the use of these travel modes will increase (H2). In contrast to cars, these modes do not provide any physical protection in a crash, and therefore have higher injury risks. It was, therefore, expected that an increase in travel, combined with travel modes with high injury risks will lead to higher road mortality - even when corrected for the travel distance - in early adolescence than in childhood (H3).

In addition to travel distances, and riskier transport modes, trip conditions may also change because of the above mentioned novelty seeking. This greater tendency in early adolescence to search for new, novel and exciting experiences, may expose youngsters to new and unfamiliar traffic situations, for which their skills may not yet be sufficiently developed. Inexperience has been shown to be an important factor in road crashes of young drivers [2], but as yet only a few studies have looked at this phenomenon for other traffic modes. In the present study, inexperience is predicted to play a role for moped riders from age 16 onwards when they can get licensed for riding a moped, and for cyclists from age 12 onwards, when youngsters start commuting to secondary schools which requires negotiating complex and unfamiliar traffic situations (H4). In epidemiological data, the role of inexperience can be identified by an initial high fatality risk per distance travelled, followed by a steady decline as experience grows [12].

In early adolescence, the detrimental effects of higher mileage, use of riskier transport modes, and inexperience may be amplified by a strong rise in sensation seeking and deliberate risk taking [11], which starts around age 10 and reaches its peak around age 16 after which it steadily declines [13]. Recent studies on brain development suggest this pattern to be the result of the way in which the structure of the adolescent brain changes as it develops. These are extremely complex processes, but in essence can be described as the ‘reward systems’ located in the limbic system becoming highly activated under the influence of puberty-related hormones and the ‘planning and control systems’ located in the prefrontal cortex, developing at a much slower pace and reaching their mature forms in one’s early 20’s [13,14]. As a result, young adolescents have difficulty controlling their impulses and are highly flexible in goal attainment, with short term gains being more attractive than long term ones, especially when peer admiration is involved [15]. These effects are stronger among males than among females [16,17], which might explain why studies on gender differences have found higher risk taking among young males compared to females [6,18,19]. Given these gender differences in sensation seeking and their impact on behaviour, fatality rates per distance for young males were expected to be higher than for young females for all travel modes (H5).

## Methods

### Data

Information on *adolescent travel* in terms of distance and travel mode by age and gender for the years 2002 to 2009 was derived from the *Dutch National Travel Statistics* (Data source CBS-OVG, IenM-MON), which contains the yearly national averages that are based on a yearly national travel survey of a representative sample of Dutch households.

To compare different causes of death, including road crashes by age and gender for the period 2002–2009, the *Dutch Mortality Record* (DMR source CBS Statistics the Netherlands) was used, which contains information on all causes of death of *Dutch citizens*, including road crashes.

Information on gender, age by year and traffic mode of *road fatalities* for the years 2002–2009 was derived from the *Dutch Road Crash Data Base*, (data source BRON/SWOV Central Bureau of Statistics [CBS], the Netherlands). This data base contains detailed information about crash circumstances, based on police records of road crashes along the entire road network in the Netherlands. The registration rates for road fatalities are satisfactory, as approximately 94% of pedestrian fatalities, 88% of cyclist fatalities, and 96% of moped rider fatalities are included in the database [20]. There is only a slight difference with the *DMR*, in that the police statistics only include information from accidents on Dutch roads, whereas the DMR also contains information on the few Dutch citizens who died in a road crash outside the Netherlands. However, these differences are too small to be of influence and are therefore not addressed in the study.

### Measures

The following measures were used in the study:

•*Road crash and road fatality*. A road crash is defined as ‘…an event on a public road that results in damage to objects and/or injury to persons and involves at least one moving vehicle, and a road fatality was defined as ‘… a person who died within thirty days from injuries sustained in a road crash’. For comparisons among the different causes of death, the Dutch Mortality Records were used and mortality was expressed as the number of fatalities per 100 000 inhabitants of that age group.

•*Natural and unnatural death*. ‘Natural death’ was defined as mortality caused by disease, and ‘unnatural death’ was defined as caused by external ‘violent’ impacts on the body leading to injuries.

•*Distance travelled* was expressed as kilometres per year per capita of that age group.

•*Road risk* was expressed as the number of road fatalities per 10^9^ kilometres.

•*Independent traffic mode* meant being in control of a vehicle as the driver instead of being a passenger. In this context, ‘walking’ is considered an independent traffic mode.

## Results

### Changing mobility patterns in early adolescence

**Figure 1:**
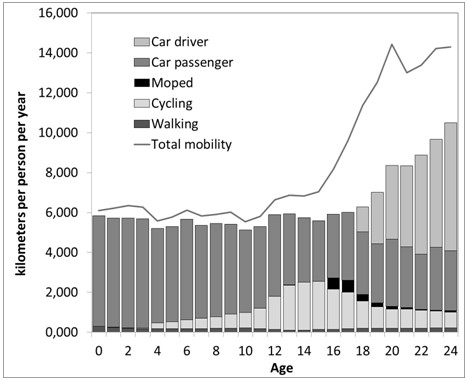
Development in mobility patterns with age, years 2002–2009 (Data source CBS-OVG, IenM-MON).

Travel per mode is presented in Figure 1, showing that up to age 16 the total distance travelled increases and that the distribution across the different transport modes changes considerably. While children up to age 11 are mainly transported by car, youngsters older than 12 travel more often independently, as cyclist and moped rider while the amount of walking kept rather constant. At age 15, youngsters travel about 2400 km as cyclists compared to 3000 km as car passengers. The analyses further showed that, with the exception of moped use, which is most popular among males, gender differences in travel patterns are only marginal. The higher use of bicycles and mopeds in combination with the lower mileage as car passenger supports the hypotheses that travel patterns change in early adolescence toward independent travel (H1) and toward more risky modes of transport (H2).

### Mortality causes in early adolescence

**Figure 2:**
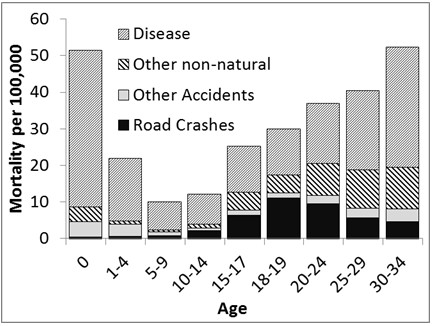
**Yearly mortality by age and cause of death in the Netherlands for the period 2002–2009 (source Dutch Mortality Records CBS/SWOV).** Note: Disease at age 0 is a factor 10 higher than presented here. For definitions of road crash and mortality see method section.

To examine adolescent road mortality from a public health perspective, Figure 2 presents the natural and unnatural mortality causes per capita and by age group. For the purpose of the present study, the original age category 15 to 19 available in the DMR, was divided into two categories for the road crash data: 15 to 17 and 18 to 19. The *Dutch Road Crash Data Base,* which contains accurate counts of the fatalities on Dutch roads by age and gender, was used to estimate the distribution of fatalities in the two age groups. The data show that while in the first decade of life, natural death dominates the mortality statistics, in the second decade injuries start to become almost as prominent a mortality cause as disease. Road mortality is responsible for a large share of that mortality, not only among the 18 to 24 year olds, the age group in which youngsters get licensed to drive cars, but also in the pre-license period. Road mortality starts to rise from age 10–14 onwards, reaching its peak in the 15 to 17 year old group. This confirms that in a late licensing country, road mortality also becomes a main cause of death among pre-license teens (H3).

### Gender differences in mortality from injuries

**Figure 3:**
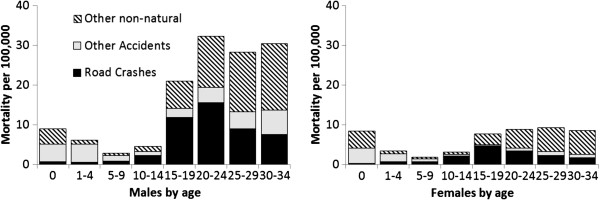
Yearly mortality for ‘unnatural death’ by age and gender per 100 000 in each age group for the period 2002–2009 (source Dutch Mortality Records CBS/SWOV).

The development of unnatural mortality by gender and age are presented in Figure 3, and shows that unnatural mortality is higher among males than among females. This difference is already visible at a very early age (1–4 years old), but becomes larger as males get older, reaching its peak around age 20–24. The development of road fatalities reflects this pattern. Up to age 5–9, road mortality is low and differs only slightly by gender. From age 10–14 it starts to rise for both sexes, but gender differences start to emerge from age 15. From age 15 onwards, road mortality of males is about a factor of three higher than that of females, indicating that already in pre-license teens, males have a higher road mortality rate that in magnitude resembles that of older males.

### Road mortality among teens by traffic role, transport mode and gender

**Figure 4:**
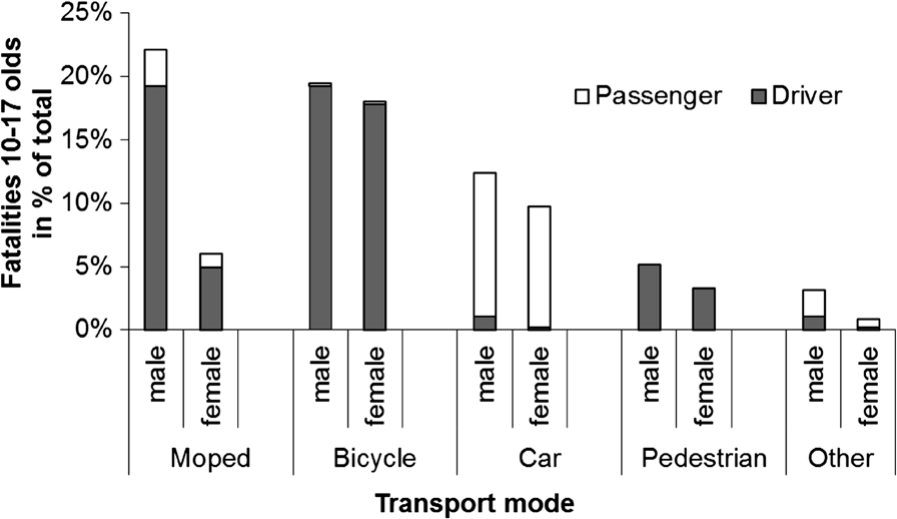
The distribution of fatalities among 10–17 year olds by transport mode and gender as a percentage of total road mortality in this age group irrespective of gender in the period 2002–2009 (data source BRON/SWOV).

In terms of traffic roles - passengers or independent travel- the crash data show that the majority lose their lives travelling independently. Only a quarter of these youngsters die as passengers in cars, whereas the majority (72%) lose their lives travelling independently as cyclists (40%), moped riders (24%) or as pedestrians (8%). There are also large gender differences (see Figure 4). First, males are overrepresented among all independent traffic roles but not in the passengers roles. Second, males are greatly overrepresented among fatally injured moped riders, whereas this is not the case for the other independent travel modes. Thus, hypothesis H5 inferring a overrepresentation of males is only confirmed for moped riders and not for the other independent travel modes.

### Road risk: road mortality corrected for exposure

**Figure 5:**
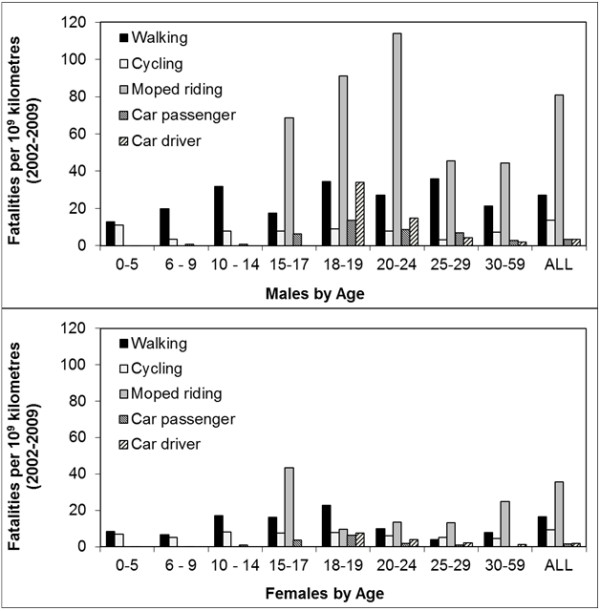
**Involvement in fatal crashes per 10**^**9 **^**kilometres by gender, transport mode and age group.** ‘ALL’ presents the average risk for all age groups (0 to 75+ of age). (Data source CBS-OVG, IenM-MON, years 2002-2009/ BRON/SWOV).

To compare the risk profiles of the different transport modes, fatality rates per distance travelled were calculated for males and females, and compared to the risk averages for the travel mode (see Figure 5). These risk averages show that, compared to the fatality risk of car passengers, the fatality risk of vulnerable road users is much higher. For cyclists this is a factor of 6, for moped riders a factor of 25, and for pedestrians a factor of 9 higher, which confirms that the earlier observed shift from being a car passenger as child to a vulnerable road user as a young adolescent, indeed implies a migration from rather safe to far riskier modes of transport (H2). This is particularly the case for the 10 to 14 year olds because of the extremely low risk of car passengers, and to some lesser extent for the 15 to 17 year olds because of the rising risk of car passengers.

Regarding the role of experience, it was hypothesized that risks for moped riders would peak around age 16 and for cyclists at the start of the secondary school (age category 10–14), which would be followed by a steady decline (H4). Figure 5 shows that the initial risk of moped riders is indeed high, but that it differs in magnitude and trajectory for females and males. The initial risk of female moped riders is only half that of males, and whereas female risk steadily declines with age, that of males steadily increases up to age 20–24 before then decreasing.

Also, cycle risk develops differently than expected. It does not show the expected peak in the age category 10–14, nor the expected gradual decrease because of growing experience in later age periods. Moreover, in contrast to moped risk, cycle risk does not differ in magnitude or in trajectory between males and females. Consequently, the large 40% share in the cycle fatalities is mainly a result of higher cycle mobility rather than of inexperience. Thus, H4 was not supported for cycling or for male moped riders. Only the risk trajectory of female moped riders is suggestive of a strong influence of inexperience.

Regarding the influence of higher sensation seeking among males, it was expected that all three non-car traffic modes would be affected, showing higher risks for males than for females. Figure 5 shows that only among moped riders do males have a convincingly higher risk than females. No gender differences were observed in cycle risk in any of the age groups. For pedestrians, gender differences become apparent from age 18 onwards. Thus the expectation that the fatality risk of males would be higher than that of females (H5) was only confirmed for moped riders and pedestrians from age 18, but not for cyclists.

## Discussion

The results confirmed that in the Netherlands road mortality among young adolescents is higher than among children and that this rise is mainly the result of male and female adolescents travelling larger distances, becoming independent road users and users of riskier traffic modes, mainly bicycles and mopeds, while walking distances do not change. This shift requires further investigation in relation to trip conditions (e.g. time of day, day of week) and the attractiveness of alternative transport modes (e.g. being a passenger of a novice driver).

The results supported the hypothesis of higher road mortality among young males, but disaggregation of the data showed this to be primarily due to a high crash risk as moped riders, where factors such as inexperience and deliberate risk taking may play an additional role. In the age category 18 to 19, the fatality risk of moped riding is about 3 times that of car drivers. In theory, this suggest that measures that encourage migration from car driving to moped riding, such as night-time and passenger restrictions, shown to be effective in reducing novice driver risks in early licensing countries [21], may have detrimental effects in countries where moped riding is an attractive alternative to car driving [2]. In the Netherlands, this impact is still small as most youngsters use bicycles instead of mopeds. But in countries with a strong ‘moped culture,’ such as Italy and Greece, these impacts may be considerable. Indeed, in cities such as Rome and Athens, more road users are killed as moped and motorcycle riders than as car occupants [22]. Studies on the effects of measures on the modal split are needed to actually assess their effects on safety.

Compared to mopeds, cycling is relatively safe, but not compared to the low risk of car passengers, especially for the 10 to 14 year olds. For the 15 to 17 year olds, the risks of cycling do not change, but the risks of car passengers do. Their passenger risk increases, probably because of this age group now being more often passengers in a car with a novice driver at the wheel, while in the younger age group there is more often an experienced driver at the wheel (see an overview of the Dutch data on passenger risks and novice drivers: [23]).

The study postulated effects on safety because of inexperience, and expected higher fatality rates per distance travelled for males compared to females. These expectations were not confirmed for cycling, as novel and unfamiliar cycle conditions did not result in higher fatality risk, and the risks of males and females did not differ, but were partially confirmed for mopeds. The unexpected finding for cycling may be related to early experience. In the Netherlands, on average children start cycling supervised by their caretakers from age 4 as part of their day-to-day trips [24]. In this process they may develop skills that protect them from harm once they start cycling solo around age 8,5 in residential areas [24] and later around age 12 in city areas. This possible protective effect of early experience should be explored in order to enhance understanding of the interactive relationship between cycling competence and exposure to risk. The relevance of such a study is growing, because of recent trends that may decrease the levels of safe practice exactly at ages in which the child’s brain is optimally ‘wired’ to learn new skills [15]. First, because of time pressure and perceived lack of safety, a growing group of parents prefers to transport their children by car rather than to accompany them on a bicycle [24]. Second, because of the low status of cycling, children from non-western origins constituting 16% of the Dutch child population [25] prefer to use other means of transport [26]. Not only would such a development affect cycling competence, it also has a negative impact on the health gains that are associated with active travel, and for cycling in particular [27].

The other finding that needs further exploration is the relatively low share of adolescent road fatalities in relation to the share of adolescents in the population. The study probably leads to an underestimate of the magnitude of the road safety problem because of this focus on road fatalities and the high physical resilience of young adolescents. A recent study of data from hospital discharges confirmed the ‘high resilience’ hypothesis, showing that for adolescents, the *injury risk* per distance travelled was the highest of all age groups, and about as high as that of the well-known high risk group of 75 and older [28], but that the proportion of seriously injured persons who died was much higher for the 75+ age group (20%) than for the age group 15 to 17 (3,5%). However, little is known about the severity and long-term consequences of these road injuries among young adolescents. The national estimate that overall 4% of injuries will result in life long disabilities [29], may not apply to this age group. Therefore, to assess the full impact of road crashes involving adolescents, further study is required into the long-term consequences of injuries.

Most likely, the studied relationship between changing mobility patterns and road mortality is not unique to the Netherlands but may also apply to other late licensing European countries. Although the role of changing mobility patterns in these EU states could not be explored because of the absence of reliable data, the fatality data in the EU also shows fatality rates rising in early adolescence with higher rates for males [30,31] and a high share (44%) of young male fatalities involving a motorized two-wheeler. Data from other European late licensing countries are thus suggestive of similar phenomena to be present, but more detailed comparisons among and between early and late licensing countries are needed to statistically test the generalizability of these results to other late-licensing countries and in addition, to assess the differential effects of licensing age on the mobility and safety of pre-license teens as well as that of teens at licensing age.

## Conclusion

Given the goal of independent mobility in early adolescence, the present study examined changing mobility patterns with age and by gender and assessed the effects on road mortality and risk in an early licensing country. The study confirmed the importance of changes in mobility patterns for understanding the rising road mortality when youngsters enter into their teens.

## Competing interests

The author(s) declare that they have no competing interests.

## Authors’ contributions

DT was the lead writer and contributed to conception and design, acquisition of data, analysis and interpretation of data, and drafting and revising the manuscript. JTS and GK supervised the study and provided assistance in analysing the data, interpreting the results and writing the final manuscript. NB provided specific knowledge on the strength and limitations of available data-bases and made substantial contributions to the acquisition and quality assurance of data as well as writing of the final manuscript. All authors read and approved the final manuscript.

## Pre-publication history

The pre-publication history for this paper can be accessed here:

http://www.biomedcentral.com/1471-2458/13/333/prepub
